# On the Treatment of Gonorrhœa

**Published:** 1885-07

**Authors:** 


ON THE TREATMENT OF GONORRHŒA. *By* J. L. MILTON, *Senior Surgeon to St. John's Hospital for Diseases of the Skin, London. Fifth edition. 8vo., cloth, pp. viii., 306. New York:* WILLIAM WOOD & CO. 1884. *Chicago:* W. T. KEENER, *96 Washington Street.*

This was the February issue of Wood's Library, and is a thorough exposition of the subject of which it treats. The following from the preface will give a good idea of the different chapters: "The following work contains, in an abridged form, the substance of the earlier editions; the papers on scalding, chordee, and gonorrhœa printed in the *Medical Times;* those on the treatment of gonorrhœa published in the *Medical Circular*, and several papers read before the Medical Society of London, and the North London and Western Medical Societies. The section on the treatment of gleet, on gonorrhœa in the female, on orchitis, and on gonorrhœal rheumatism, have been revised and amplified. Those on gonorrhœal affections of the heart and pericardium, the peritoneum and pleura, dura mater and sheath of the spinal cord, on gonorrhœal pyæmica, pyelitis, etc., are now added for the first time." Matters pertaining to treatment are discussed at length, and a comparative study of the different therapeutical means is attempted, but the writer's experience seems to have been so vast that he has come to give up any sanguine expectations from remedial agents. The vast amount of quotations and references unfit the book for ordinary practitioners, but render it doubly valuable to a teacher or a specialist.

